# A rare case of biatrial myxoma in an 11-year-old girl patient with thromboembolic stroke: A case report

**DOI:** 10.1016/j.ijscr.2025.111311

**Published:** 2025-04-17

**Authors:** Navy Laksmono, Chrisna Wariz Tansa, Bela Ita Karina, Nia Anestya, Hasrayati Agustina

**Affiliations:** a Cardiothoracic Surgery Division, Department of Surgery, Faculty of Medicine, Universitas Padjadjaran – Dr. Hasan Sadikin General Hospital, Bandung, Indonesia; b General Surgery Division, Department of Surgery, Faculty of Medicine, Universitas Padjadjaran – Dr. Hasan Sadikin General Hospital, Bandung, Indonesia; c Department of Anatomical Pathology, Faculty of Medicine, Universitas Padjadjaran – Dr. Hasan Sadikin General Hospital, Bandung, Indonesia

**Keywords:** Cardiac myxoma, Biatrial myxoma, Cardiac tumor, Case report

## Abstract

**Introduction:**

Cardiac myxomas (CM) are benign primary tumors typically found in the left atrium, but biatrial myxomas are exceptionally rare, comprising only 3–5 % of cases. This report highlights the rarity of biatrial myxomas in a young patient, the importance of early recognition due to the risk of thromboembolic events, which can lead to stroke. Excellent surgical intervention is needed to prevent CM recurrences.

**Case presentation:**

An 11-year-old girl presented with sudden onset of left-sided hemiparesis, aphasia, right-sided facial drooping, cephalgia, palpitations, nausea, and intermittent chest pain over the past two months. Laboratory examination revealed anemia and elevated D-Dimer. Echocardiography and cardiac CT demonstrated large masses in the right and left atrium, suggesting myxomas. The patient was diagnosed with biatrial myxomas complicated by thromboembolic stroke.

**Clinical discussion:**

Complete mass evacuation was performed through median sternotomy. Histopathology examination confirmed CM.

**Conclusion:**

This case emphasizes the importance of early detection, detailed examination and surgical intervention of biatrial myxomas, especially in young patients, to prevent and manage life-threatening thromboembolic complications and recurrence of CM. Timely intervention is crucial for ensuring favorable outcomes.

## Highlights

• Cardiac myxomas (CM) are common benign tumors that primarily occur in the left atrium but can rarely develop in both atria—known as biatrial myxomas.
• The clinical presentation of CM depends on their location, size and mobility and is typified by the triad of intracardiac obstruction
• Cerebral embolisation is relatively common in CM, leading to ischemic stroke and cerebral aneurysm formation.
• Large resection of the myxoma pedicle or stalk is essential to prevent recurrence and the subsequent need for reoperation.
• Biatrial myxoma in children is a rare condition which needs early diagnosis and surgical excision to prevent complications and improve outcomes.

## 1 Introduction

Cardiac myxomas (CM) are common benign tumors, with an incidence of 0.0017 % [1]. They primarily occur in the left atrium but can rarely develop in both atria—known as biatrial myxomas, which account for 3–5 % of all CM cases [2]. These tumors are most often diagnosed in individuals aged 30–60, with a female predominance [3]. CM can cause a variety of symptoms depending on their size, location, and mobility. A significant concern is the potential for embolic events, occurring in 20–45 % of patients [4], which can lead to stroke or peripheral embolism [5]. The risk is particularly high in tumors with a friable structure. This case highlights the need for better awareness of embolic events caused by atrial myxomas in young patients. Once diagnosed, surgical excision of the tumor is crucial. We present a successful surgery to remove a large biatrial myxoma in an 11-year-old girl with thromboembolic stroke.

## 2 Case report

An 11-years-old girl patient presented to the hospital following sudden onset of left- sided hemiparesis, aphasia and right-sided drooping face 2 h before admission. The patient also complains of cephalgia, palpitations, nausea and vomit. There has been intermittent chest pain for the past two months. There is no history of fever, convulsion, loss of consciousness and other familial disease. On physical examination, the patient was tachycardic with regular rhythm and no abnormal sound. No edema and rashes were found. Neurological examination showed loss of motoric function on the left side extremities.

**Fig. 1:**
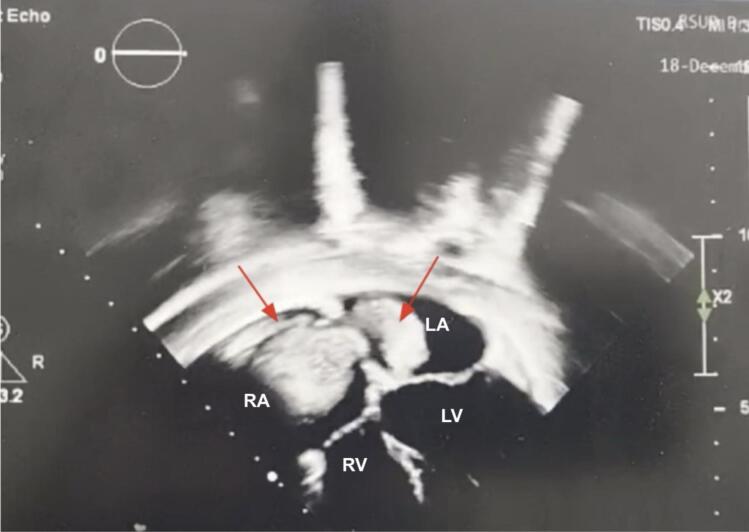
Preoperative echocardiography showing mass (red arrows) filling RA and LA which connected via atrial septum. LA (left atrium); RA (right Atrium); LV (left ventricle); RV (right ventricle).

**Fig. 2:**
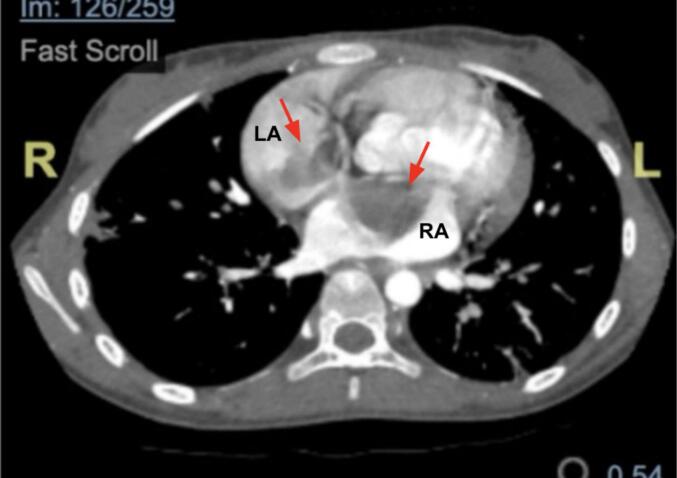
Cardiac CT showing dilated RA and LA with hypodense lesions (red arrows) measuring approximately 3,67 × 4,0 cm in the RA and 2.49 × 3.73 cm in the LA. LA (left atrium); RA (right Atrium).

Initial laboratory examination revealed anemia and elevated D-Dimer (3 times upper normal limit). Prothrombin time and activated partial thromboplastin time were normal. Electrocardiography demonstrated sinus tachycardia, ST depression with abnormal T Wave. Due to these findings, additional workup was needed to identify the etiology. An echocardiography showed intracardiac mass at right and left atrium (Fig. 1). Cardiac CT revealed dilated right atrium (RA) and left atrium (LA) with hypodense lesions, well-defined borders, irregular edges, the largest measuring approximately 3,67 × 4,0 cm in the RA and 2.49 × 3.73 cm in the LA (Fig. 2). Given the patient's demographic characteristic and no sign of metastatic disease, cardiac myxoma was suspected and urgent operation was scheduled.

**Fig. 3:**
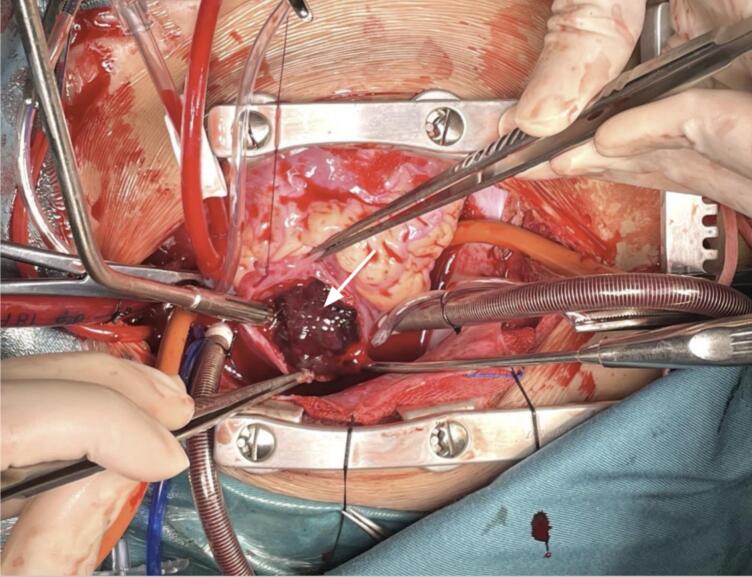
Intraoperative finding: gelatinous-like mass (white arrow) filling the whole right atrium.

Median sternotomy was performed following cannulation through ascending aorta, as well as superior vena cava and inferior vena cava. After aortic cross-clamping, cardiopulmonary bypass was initiated and the heart was arrested with del Nido cardioplegia. The right oblique atriotomy was performed and showed a mass filling the whole right atrium (Fig. 3). Atrial septum then excised in order to expose and remove the left mass. The left intracardiac mass was found attached to mitral annulus. After complete evacuation of the mass, the resulting atrial septal defect was closed with a pericardial patch.

**Fig. 4:**
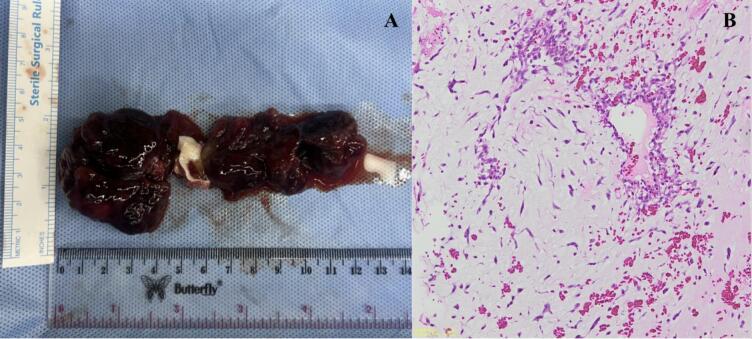
**A.** Gross Examination: benign jelly-like masses, measuring 6 × 5 × 3 cm, and 6 × 2.5 × 2.5 cm with connecting stalk, suggesting myxoma. **B.** Microscopic examination: Stellate cell within myxoid stroma, complex structures resembling rings around blood vessels.

**Fig. 5:**
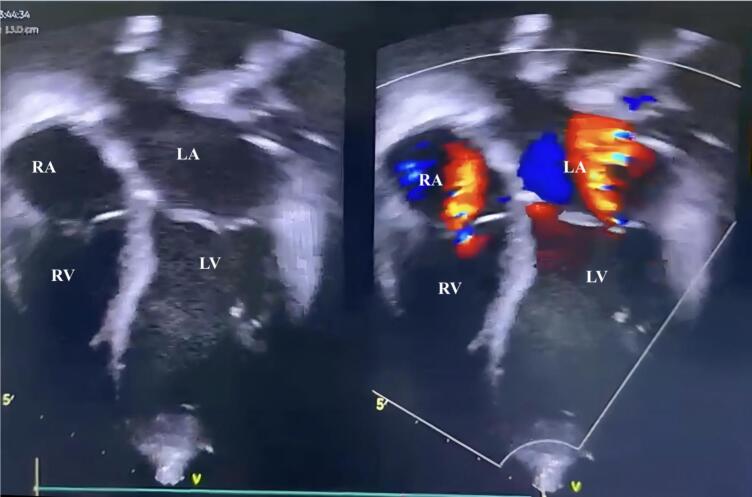
Postoperative echocardiography: complete removal of the tumor, no residual shunt from atrial septum. LA(left atrium); RA (right Atrium); LV (left ventricle); RV (right ventricle).

The macroscopic examination demonstrated benign jelly-like masses, suggesting myxoma measuring 6 × 5 × 3 cm, and 6 × 2.5 × 2.5 cm (Fig. 4A). The microscopic examination showed stellate cells within myxoid stroma, complex structures resembling rings around blood vessels, confirming the diagnosis of CM (Fig. 4B). The postoperative course proceeded without any complications and the patient displayed normal sinus rhythm without the need of a pacemaker. The patient was extubated 2 h after surgery and stayed in the ICU for 2 days. Postoperative echocardiography shows complete removal of the tumor (Fig. 5) and no residual shunt from atrial septum. The patient was discharged on postoperative day 7. Upon routine follow-up in outpatient clinics at 2 weeks later the patient showed improvement in mobilization and there is no complaint related to cardiac symptom or postoperative complications. The patient undergoes a physical rehabilitation program to overcome the stroke sequelae. 2 months later, an echocardiography examination was performed and there is no sign of CM recurrences, the patient can walk without any assistance and is able to do activity daily living.

## 3 Discussion

CM is a common benign primary intracardiac tumor with an incidence rate of 0.0017 % and can affect any chamber of the heart [1]. The most common affected side including LA (60 %–88 %); RA (4 %–28 %); LV (8 %); and RV (3 %–4 %), with sizes ranging from 0.18 cm to 6 cm [7,9]. Studies have reported that approximately 64 % of sporadic myxomas occur in females [ 8]. Myxomas are among the more prevalent benign cardiac tumors in children, accounting for 21.5 % of all benign cardiac tumors in this population [9]. Biatrial myxoma in children is an exceptionally rare, comprising only about <2.5 % of all cases of CM [6,7]. Due to their rarity, specific prevalence data for biatrial myxomas in pediatric patients are limited.

Regarding its origin, CM has a predilection for limbus fossa ovalis of the interatrial septum [1]. The clinical presentation of CM depends on their location, size, mobility and is typified by the triad of intracardiac obstruction: obstructive symptoms, embolic phenomena, and constitutional symptoms [10] Cerebral embolisation is relatively common, leading to ischemic stroke and cerebral aneurysm formation [1,3]. There were a large number of patients who did not resort to consultations until suffering from serious complications like heart failure or cerebral embolism [11]. Our patient was admitted to hospital due to neurologic manifestations (left-sided hemiparesis, aphasia, right-sided facial drooping and cephalgia). The diagnostic work up towards myxoma was achieved due to suspicious history of palpitations and intermittent chest pain, presence of ST depression and abnormal T wave in electrocardiography, abnormal laboratory result (high D dimer 3 times upper limit), and presence of mass in echocardiography which further confirmed with CT cardiac,

CM are derived from multipotent mesenchymal cells, which develop into a gelatinous mass. These are generally solitary and pedunculated, often attached to the atrial septum via a stalk [12,13]. The etiology remains unspecified but the CM harbor similar protein expression described in endocardial-mesenchymal transformation of the endocardial cushion [14]. Most of them are sporadic, but about 10 % are associated with the Carney complex, an autosomal dominant disorder caused by mutations in protein kinase A regulatory subunit 1 alpha (PRKAR1A) [15]. Our patient did not have any conditions consistent with Carney complex.

Echocardiography is the critical investigation for the diagnosis of CM. Transthoracic echocardiography (TTE) is the most practical investigation and plays an extremely important role in the diagnosis and follow up of myxoma, its sensitivity can reach to 100 % [11,13]. The diagnostic approach in this case was thorough, incorporating both imaging and clinical evaluation. Echocardiography provided the critical diagnosis, revealing intracardiac masses in both atria. Cardiac CT further delineated the size, location, and characteristics of the masses, confirming the diagnosis of biatrial myxoma and crucial for surgical planning. The diagnosis was further corroborated by the macroscopic pathology findings of a benign, jelly-like mass typical of myxoma.

According to Jones and colleagues, the surgical approach for atrial myxomas should allow minimal manipulation of the tumor, provide adequate exposure for complete resection of the mass, allow inspection of all four heart chambers, and be safe and efficacious [3]. A large resection of the myxoma pedicle or stalk is essential to prevent recurrence and the subsequent need for reoperation [3]. For biatrial myxoma, RA incision and atrial septal incision have good exposure. To reduce the possibility of partial recurrence, normal tissue around the tumor should also be removed at least 0.5–1 cm, valve forming or replacement could be implemented after operation. Myxomas are treated by surgical resection, harboring an overall good prognosis [14]. In our patient, with complete resection of the tumor, we find no sign of CM recurrence up to 2 months after surgery.

Case reports and studies from other centers emphasize the rarity of biatrial myxomas, particularly in pediatric patients and their association with thromboembolic complications, including stroke. The embolic risk is influenced by the size, mobility, and friability of the tumor, as well as the hypercoagulable state induced by the tumor itself. In pediatric patients, the presentation of a thromboembolic stroke should raise suspicion for underlying cardiac pathology, such as myxoma, particularly in the absence of traditional vascular risk factors. Early recognition and imaging, including echocardiography, are essential to establish the diagnosis and facilitate timely surgical intervention to prevent further embolic events [11].

## 4 Conclusion

In conclusion, biatrial myxoma in children is a rare condition with varying clinical manifestations, making early detection challenging. Symptoms can range from asymptomatic to severe embolic events, as seen in this case. Awareness and a high index of suspicion for cardiac myxomas in young patients with unexplained stroke or embolism are crucial. Early diagnosis and prompt surgical excision are essential to prevent complications and improve outcomes. Our patient illustrates the importance of thorough examination and the significance of surgical approach in treating CM.

## Consent

Written informed consent was obtained from the patient's parents about these case reports writing and publishing. They understood well and gave consent. A copy of the written consent is available for review by the Editor-in-Chief of this journal on request.

## Guidelines

We followed The SCARE 2023 Guideline: Updating Consensus Surgical Case Report (SCARE) Guidelines [16].

## Ethical approval

As it is a case report, ethical approval is exempted by our institution.

## Funding

This research did not receive any specific grant from funding agencies in the public, commercial, or not-for-profit sectors.

## Author contribution

Conception and design of study, acquisition of data, analysis and interpretation of data, drafting the manuscript, revising the manuscript critically for important intellectual content, approval of the version of the manuscript to be published.

## Guarantor

Navy Laksmono acts as the author and the guarantor of this article. Author is fully responsible for the work, the conduct of the study, data accessibility and decision to publish.

## Declaration of competing interest

The author(s) declared no conflict of interest regarding the publication of this article.

## Data Availability

All supporting data are available within this study.
